# A Logistic Regression Model for Predicting the Risk of Subsequent Surgery among Patients with Newly Diagnosed Crohn’s Disease Using a Brute Force Method

**DOI:** 10.3390/diagnostics13233587

**Published:** 2023-12-03

**Authors:** Kohei Ogasawara, Hiroto Hiraga, Yoshihiro Sasaki, Noriko Hiraga, Naoki Higuchi, Keisuke Hasui, Shinji Ota, Takato Maeda, Yasuhisa Murai, Tetsuya Tatsuta, Hidezumi Kikuchi, Daisuke Chinda, Tatsuya Mikami, Masashi Matsuzaka, Hirotake Sakuraba, Shinsaku Fukuda

**Affiliations:** 1 Department of Gastroenterology and Hematology, Hirosaki University Graduate School of Medicine, Hirosaki 036-8562, Japan; o.kohe@hirosaki-u.ac.jp (K.O.);; 2 Department of Medical Informatics, Hirosaki University Hospital, Hirosaki 036-8563, Japan

**Keywords:** Crohn’s disease, surgery, prediction, brute force method, newly diagnosed

## Abstract

Surgery avoidance is an important goal in Crohn’s disease (CD) treatment and predicting the risk of subsequent surgery is important to determine adequate therapeutic strength for patients with newly diagnosed CD. Herein, we aimed to construct a prediction model for the risk of subsequent surgery based on disease characteristics at the patients’ initial visit. We retrospectively collected disease characteristic data from 93 patients with newly diagnosed CD. A logistic regression model with a brute force method was used to maximize the area under the receiver operating characteristic curve (auROC) by employing a combination of potential predictors from 14 covariates (16,383). The auROC remained almost constant when one to 12 covariates were considered, reaching a peak of 0.89 at four covariates (small-bowel patency, extensive small-bowel lesions, main lesions, and the number of poor prognostic factors), and it decreased with increasing covariate size. The most significant predictors were small-bowel patency, extensive small-bowel lesions, and age or major lesions. Therefore, this prediction model using covariates may be helpful in determining the likelihood that a patient with newly diagnosed CD will require surgery, which can aid in appropriate treatment selection for high-risk patients.

## 1. Introduction

Inflammatory bowel disease (IBD), including Crohn’s disease (CD) and ulcerative colitis, is a chronic inflammatory disease of the gastrointestinal tract. CD is a pan-enteric chronic inflammatory disease characterized by granulomatous inflammation of all layers and skipped lesions. The disease behavior of CD is defined according to the Montreal Classification [1] as inflammatory (B1), stricturing (B2), or penetrating (B3), and disease location is defined as ileal (L1), colonic (L2), ileocolonic (L3), or upper gastrointestinal tract (L4). Most patients with newly diagnosed CD exhibit the B1 phenotype. However, within 20 years after diagnosis, it progresses to the B2 or B3 phenotype in 50–88% of patients [2,3,4,5]. Furthermore, approximately half of the patients require surgery within 10 years of diagnosis, and approximately the same number of patients experience relapse after 10 years [6]. In the last few decades, early interventions with immunomodulators (IMs) and biologics (BIOs), such as anti-tumor necrosis factor-α, anti-interleukin (IL)-12/23, and anti-a4b7 integrin antibodies, have been shown to prevent severe complications, such as hospital admission [7,8] and surgery [7,8,9].

However, the prediction of the risk of subsequent surgery in patients with newly diagnosed CD is important for determining adequate therapeutic strength and providing a higher quality of life. The cumulative probability of surgery in CD has been evaluated in several population-based cohorts [10,11,12,13]. Furthermore, a number of clinical risk factors have been associated with an increased need for surgery. In adult patients, young age at diagnosis is a prognostic factor for surgery [11,12,14]. Disease located in the small bowel (L1 and L3 phenotypes) has been consistently identified as an independent risk factor for surgery in adult populations [10,11,15,16,17,18]. Moreover, the B2 and B3 phenotypes at diagnosis are one of the most important factors associated with the need for surgery [10,11,18,19]. Particularly, the B2L3 phenotype, an ileocolonic type with major lesions (longitudinal ulcer and/or cobblestone appearance) in the distal ileum, shows an approximately 40% higher surgery rate at 5 years [20]. Additionally, Lazarev et al. showed that the additional presence of jejunal disease significantly increases the risk for stricturing behavior or multiple abdominal surgeries in patients with ileal (L1 or L3 phenotype) disease site compared to those having the ileal site without any proximal disease [21]. This result highlights the importance of extensive small-bowel lesions as a risk factor for surgery. Upper small-bowel lesions (L4 phenotype) are also indicative of higher surgical risk [21,22,23,24,25,26], although colonic disease (L2 phenotype) is protective against major surgery [26,27]. Moreover, anal lesions (complex perianal fistula [28,29], anal-canal stricture [30], etc.) are risk factors for permanent stoma. A Belgian study demonstrated that body weight loss (>5 kg; hazard ratio: 1.67; 95% confidence interval (CI): 1.14–2.45) at diagnosis was independently associated with time to development of severe disease [31]. Additionally, smoking is associated with the development of strictures or fistulas [32], a higher relapse rate [33], and increased risk of surgery, including re-operation [34].

Currently, the treatment for CD comprises three approaches: a ‘step-up’ approach, which is the traditional sequential treatment starting with 5-aminosalicylates; a ‘top-down’ approach [35], which is based on the initial use of BIOs; or an ‘accelerated step-up’ approach, which involves early use of IMs and/or BIOs in patients with B1 phenotype who have risk factors for the development of B2 and/or B3 phenotype. Based on a meta-analysis of 22 population-based cohort studies, the 1-, 5-, and 10-year risk of surgery in patients with newly diagnosed CD before 2000 was 23.6%, 35.7%, and 46.5%, respectively. However, presently, the 1-, 5-, and 10-year risk of surgery is 12.3%, 18%, and 26.2%, respectively, in patients diagnosed in the 21st century [36]. Optimization of the treatment strategy for patients with newly diagnosed CD or identification of high-risk patients who require a ‘top-down’ or an ‘accelerated step-up’ approach is necessary to prevent progression and avoid poor prognosis. However, accurate prediction of the prognosis of each patient is difficult due to the varied course of CD.

A binary logistic regression model is primarily utilized to predict the emergence of a pathological state or an adverse condition, employing clinical or surveillance datasets. Although stepwise selection techniques are commonly applied to identify the predictor or the amalgamation of covariates [37,38,39], they do not invariably guarantee an optimal predictor yielding maximal model performance. However, a meticulous brute force approach as the predictor selection method represents a viable strategy for optimizing model performance. In computer science, the brute force method is a widely used problem-solving technique that involves systematically checking all possible candidates to determine whether they satisfy the given problem statement or not. The method is used when the problem size is limited, and when the simplicity of implementation is more important than processing speed.

Therefore, in this study, we aimed to optimize the treatment strategy for patients with newly diagnosed CD using logistic regression analysis with a brute force method to determine the risk of surgery at diagnosis.

## 2. Materials and Methods

### 2.1. Preparation of Dataset and Patients

**Figure 1:**
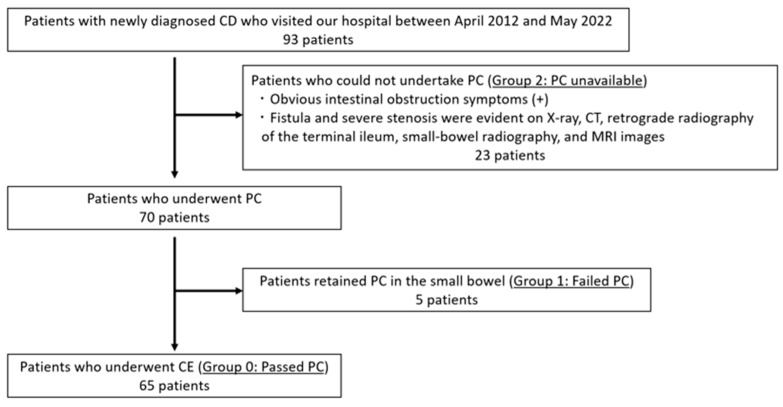
Flowchart for evaluation of small-bowel patency. CD: Crohn’s disease, PC: patency capsule, CE: capsule endoscopy.

This retrospective study included 93 patients with newly diagnosed CD who visited the Hirosaki University Hospital between April 2012 and May 2022. In patients who had no obvious luminal stenosis on radiological examinations (e.g., abdominal X-ray inspection, computed tomography, retrograde radiography of the terminal ileum, small-bowel radiography, etc.), small-bowel patency was confirmed using patency capsules (PC; Medtronic, Minneapolis, MN, USA) before the capsule endoscopy (CE) procedure. PillCam^TM^SB3 (Medtronic) was used in all adaptive patients. Regarding small-bowel patency, patients were classified into three groups: those who were able to confirm the small-bowel patency using PC (Group 0: passed PC), those who had no obvious luminal stenosis on a radiological examination but had a retained PC in the small bowel (Group 1: failed PC), and those with obvious luminal stenosis on a radiological examination (Group 2: PC unavailable) (Figure 1). Table 1 and Table S1 show the patient characteristics.

We identified the following eight items as poor prognostic factors of CD: young onset, ileocolonic type, major lesions (longitudinal ulcer and/or cobblestone appearance), extensive small-bowel lesions, upper small-bowel lesions, anal lesions, body weight loss, and smoking. Subsequently, we defined the number of items possessed by the patients with newly diagnosed CD as the ‘poor prognosis score’ (0–8). Patients aged < 18 years were defined as young onset. A variety of perianal manifestations occur in patients with CD, including perianal skin lesions (anal skin tags, hemorrhoids), anal canal lesions (anal fissures, anal ulcers, anorectal strictures), perianal fistulas and abscesses, rectovaginal fistulas, and cancer [40,41]. In this study, anal lesions included perianal fistulas or perianal abscesses detectable by computed tomography or magnetic resonance imaging. Extensive small-bowel lesions were defined as per the following three requirements: (1) (CE findings) the existence of ulcerations spreading to three-fourths or more of the upper and lower jejunum, and upper and lower ileum; (2) (CE findings) the existence of main lesions spreading to two-fourths or more of the upper and lower jejunum, and upper and lower ileum; and (3) (radiological findings) the existence of main lesions spreading over 40 cm. Moreover, the upper small-bowel lesions were defined as ulcerations spreading to two-thirds or more of the duodenum and the upper and lower jejunum. Ulceration included small, irregular, circular, linear, and longitudinal ulcers, and cobblestone appearance.

The disease characteristics observed during the initial visit were recorded, along with labels indicating whether subsequent surgery was required (presence (1) or absence (0)), and these are presented in the second column of Table 1. No statistical ranking of covariates was conducted prior to model development (Table 2).

### 2.2. A Logistic Regression Model Using Brute Force Predictor Selection

A logistic model is a statistical model that assesses the probability of an event occurring by considering the log odds for the event as a linear combination of one or more independent variables. In regression analysis, logistic regression [42] is the process of estimating the parameters of the logistic model. The Python programming language has been established as one of the most popular languages for scientific computing; scikit-learn is a Python module that integrates a wide range of state-of-the-art machine learning algorithms. It has a minimal dependency and is distributed under a simplified BSD license, encouraging its use in both academic and commercial settings. The source code, binaries, and documentation are available from http://scikit-learn.sourceforge.net (accessed on 3 October 2022).

Logistic regression modeling was used to maximize the area under the receiver operating characteristic curve (auROC) by employing potential predictor combinations derived from the 14 covariates. The iterations spanned from 1 combination of 14 to 14 combinations of 14, resulting in a total of 2^14^ − 1 or 16,383 possible combinations. Subsequent to the identification of the combination, the mean auROC, ascertained from ten iterations of three-fold cross-validation, was computed to evaluate the model’s performance. The processing for model construction and evaluation was implemented using scikit-learn, a Python library [42].

### 2.3. Statistical Analyses

Continuous or discrete covariates are expressed as averages ± standard deviations (SD); the differences in means between those with or without a second injury were assessed using the Student’s or Welch’s *t*-test. Nominal covariates are expressed as checked counts or frequency; the differences between those with or without a second injury were evaluated using Fisher’s exact test. Differences in the maximal auROC among the selected covariates were assessed using Tukey’s multiple comparison test. Survival analysis for each predefined outcome was performed using the Kaplan–Meier curve, and the log-rank test was computed. Statistical significance was set at *p* < 0.05. All statistical analyses were performed using IBM SPSS Statistics for Windows, Version 21.0 (IBM Corp, Armonk, NY, USA).

## 3. Results

### 3.1. Patient Characteristics

Patient clinical and demographic characteristics are presented in Table 1. The study included 93 patients (69 males and 24 females) with a mean age of 26 (8–67) years. The disease locations were as follows: 18 had the ileal type, 54 the ileocolonic type, and 21 the colonic type. The disease behaviors were as follows: 50 had the non-stricturing and non-penetrating (B1) phenotype, 35 the stricturing (B2) phenotype, and eight the penetrating (B3) phenotype. The presence of poor prognostic factors were as follows: 22 had young onset, 65 had major lesions, 24 had upper small-bowel lesions, 32 had extensive small-bowel lesions, 31 had anal lesions, 73 had body weight loss, and 18 had smoking. The small-bowel patency classification groups were as follows: 65 were in Group 0 (passed PC), 5 were in Group 1 (failed PC), and 23 were in Group 2 (PC unavailable). Moreover, 14 of 93 patients (15%) required subsequent surgery. Table S1 shows the patient characteristics of the 14 patients who required surgery.

### 3.2. Univariate Comparison of Covariates with or without Subsequent Surgery

The results of a univariate comparison of two continuous covariates and one discrete covariate (expressed as mean ± SD), as well as 12 nominal covariates (expressed as checked counts/frequency), between patients with CD with and without subsequent surgery, are presented in Table 2. Statistically significant differences were observed for two continuous covariates (age (*p* = 0.038) and small-bowel patency (*p* < 0.001)) and three nominal covariates (ileal type (*p* = 0.005), major lesions (*p* = 0.009), and extensive small-bowel lesions (*p* = 0.015)).

**Figure 2:**
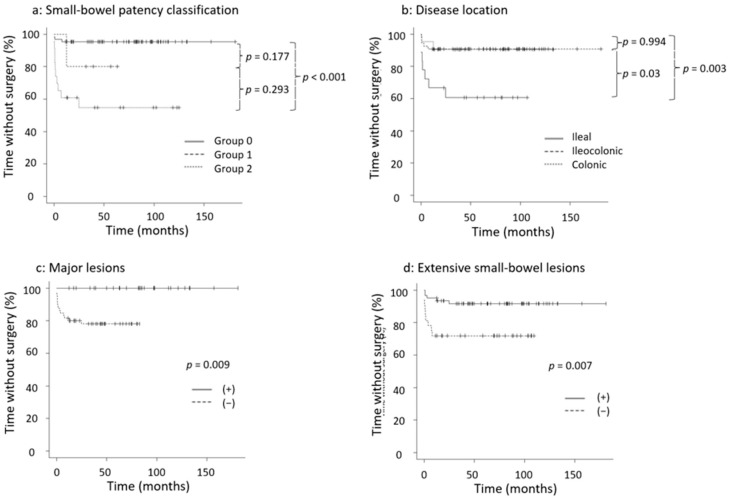
Kaplan–Meier analysis of survival without surgery. (**a**) Small-bowel patency classification, (**b**) disease location, (**c**) major lesions, (**d**) extensive small-bowel lesions. Kaplan–Meier curves were created using the log-rank test. Group 0: passed PC, Group 1: failed PC, Group 2: PC unavailable. PC: patency capsule.

Kaplan–Meier analysis of survival without surgery based on four factors (small-bowel patency classification, disease location, major lesions, and extensive small-bowel lesions) is shown in Figure 2. In the 14 patients who underwent surgery, the median time from diagnosis to surgery was 40 days, and 12 patients required surgery within 1 year of diagnosis. The log-rank test was used to evaluate the time to surgery for each factor, which was significant in predicting the risk of surgery: between Groups 0 and 2 of the small-bowel patency classification (*p* < 0.001), between ileal and colonic types (*p* = 0.003), major lesions (*p* = 0.009), and extensive small-bowel lesions (*p* = 0.007) (Figure 2).

### 3.3. The Maximal auROC against Selected Covariates

**Figure 3:**
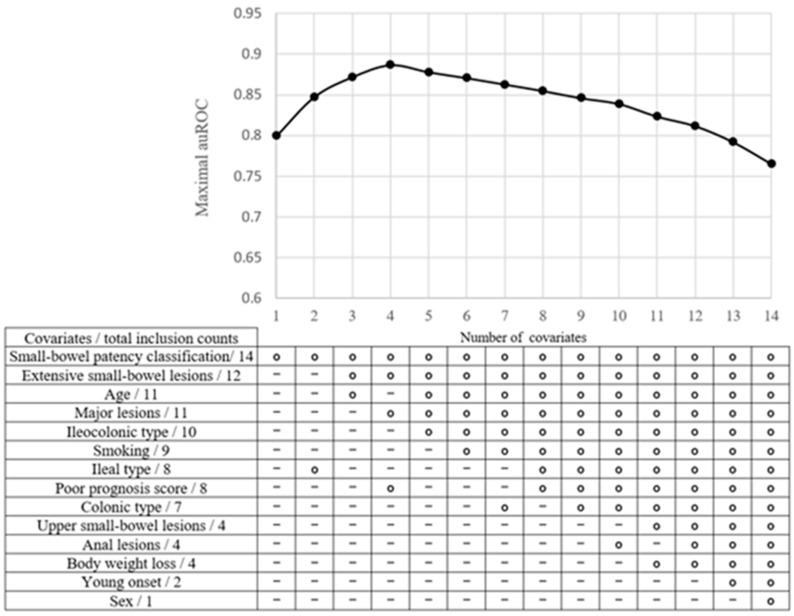
The maximal validation area under the receiver operating characteristic curve (auROC) for subsequent surgery among patients with newly diagnosed Crohn’s disease against selected covariates. In the upper panel, the maximal validation auROC for subsequent surgery against the best combination of predictors is determined via a brute force approach ranging from one to 14 covariates. In the lower panel, the covariates determined via a brute force method are sorted in descending order of total inclusion counts as predictors. The open circles in each column denote the optimal covariates chosen from 1 combination of 14 to 14 combinations of 14. A “-” symbol denotes a covariate not selected as a predictor.

The maximal validation auROC curve for subsequent surgery in patients with newly diagnosed CD was assessed against the selected covariates (Figure 3). The upper panel illustrates the maximal validation auROC for subsequent surgery against the best combination of predictors determined using a brute force approach ranging from one to 14 covariates. The lower panel depicts the covariates determined using the brute force method sorted in a descending order of total inclusion counts as predictors. As depicted, the highest validation auROC was achieved with the combination comprising 4 covariates, encompassing small-bowel patency, extensive small-bowel lesions, primary lesions, and poor prognosis score, whereas a “-” symbol indicates that a covariate was not selected as a predictor. The auROC remained practically constant when one to 12 selected covariates were considered, reaching a peak of 0.89 with four covariates, which comprised small-bowel patency, extensive small-bowel lesions, major lesions, and poor prognosis score, and decreased as the selected covariate numbers increased. The three most significant predictors as per total inclusion counts were small-bowel patency, extensive small-bowel lesions, and age or major lesions.

### 3.4. Rates of Each Small-Bowel Patency Classification Group with or without Surgery and Study Outcome

**Figure 4:**
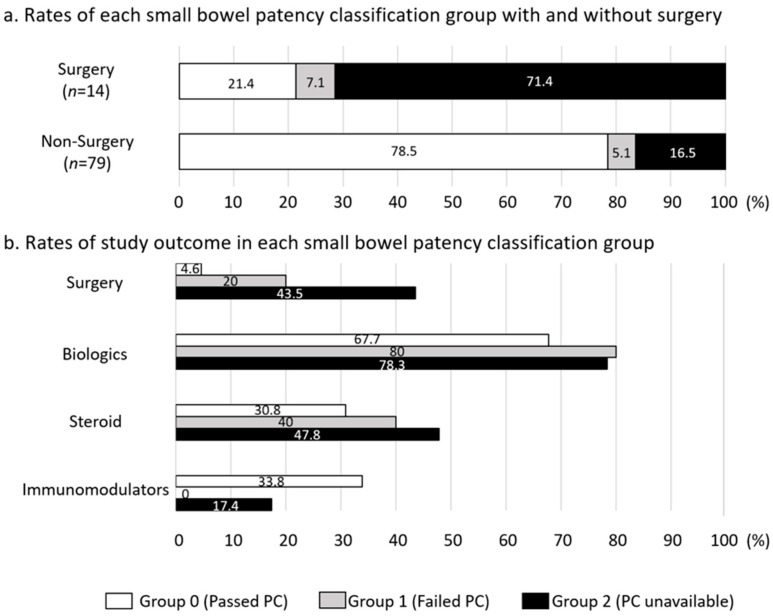
Rates of each small-bowel patency classification group with or without surgery and study outcome. (**a**) Rates of each small-bowel patency classification group with or without surgery, (**b**) rates of study outcome in each small-bowel patency classification group. The group is expressed by the column composed of white (Group 0: passed PC), light gray (Group 1: failed PC), and black (Group 2: PC unavailable). PC: patency capsule.

Figure 4 presents the rates of each small-bowel patency classification group with or without surgery and the study outcome. The group of patients with surgery had higher rates of Groups 2 (PC unavailable) and 1 (failed PC) compared to the patients without surgery group (Figure 4a). As shown in Figure 4b, Groups 1 and 2 had higher rates of surgery and utilization of BIOs and steroids when compared to Group 0. Group 0 had higher rates of IM utilization compared to Groups 1 and 2, contrary to the lower rates of surgery and BIOs and steroid utilization. These results show that Group 0 patients tended to use IMs alone without both of BIOs and steroids.

## 4. Discussion

The ultimate therapeutic goal for patients with CD is to avoid intestinal surgery and subsequent short bowel syndrome. Early ‘top-down’ therapeutic approaches should be used to prevent disease progression, which leads to poor prognosis and surgery. Thus, predicting the risk of subsequent surgery among patients with newly diagnosed CD is important for determining the appropriate therapy. Although there are several reports on the clinical factors for predicting the risk of surgery among patients with CD, accurate prediction of prognosis in patients with newly diagnosed CD remains difficult.

Herein, we developed a logistic regression model using a brute force method to predict subsequent surgeries for patients with newly diagnosed CD. The brute force method is a straightforward approach to problem solving that involves systematically checking all possible solutions or combinations without utilizing any specific optimization or heuristics. This involves exhaustively trying every possible option or input to find the desired solution. Essentially, the brute force method works by generating and evaluating all potential solutions individually until the correct solution is found or all possibilities have been examined.

Recently, Dias et al. showed a decision-tree-based approach for estimating prognosis concerning the risk of disability, surgery, and re-operation in patients with CD [43]. Additionally, Yao et al. identified disease behavior, smoking, body mass index, C-reactive protein level at diagnosis, previous perianal or intestinal surgery, maximum bowel wall thickness, use of BIOs, and exclusive enteral nutrition as independent significant factors associated with early intestinal surgery using multivariate logistic regression analysis [44]. To our knowledge, this is the first study to use a brute force method to identify the combination of predictors of subsequent surgery for patients with newly diagnosed CD that maximized the auROC. Our results revealed the following four covariates as predictors of the requirement for subsequent surgery: small-bowel patency, extensive small-bowel lesions, major lesions, and poor prognosis score.

In the past, it was difficult to evaluate whole small-bowel lesions of CD precisely. Currently, balloon-assisted endoscopy (BAE), including single-balloon and double-balloon enteroscopy, and CE are also used to evaluate lesions in the whole small bowel. The appearance of BAE has revolutionized small-bowel examinations due to its application for the histological diagnosis and the treatment of CD-associated small intestinal strictures (e.g., endoscopic balloon dilatation). Although CE cannot be used for histological diagnosis and treatment, it can be used with relatively minimal invasion in cases with confirmed small-bowel patency. Additionally, CE has equal or higher accuracy than other diagnostic modalities in patients with established CD [45]. A recent meta-analysis showed significant increase in the diagnostic accuracy with ileocolonoscopy (CS) compared with small-bowel barium radiography (38%; 95% CI, 22–54%; *p* < 0.00001) and computed tomography enterography (32%; 95% CI, 16–47%; *p* < 0.0001), but not with CS (13%; 95% CI, −1–26%; *p* = 0.07) or magnetic resonance enterography (−6%; 95% CI, −30–19%; *p* = 0.65) in patients with established CD. It has been demonstrated that achieving deep remission with mucosal healing (MH) is important for avoiding surgeries in patients with CD [46]. Although CS is considered the gold standard for determining MH in CD [45,47,48,49], there are several reports on the usefulness of CE in evaluating small-bowel lesions of CD. However, a major complication of CE in patients with CD is the retention of CE due to small-bowel stenosis [50]. Consequently, the American and European guidelines recommend using PC or small bowel imaging before CE if a small-bowel stricture is suspected in patients with established CD [51,52]. Recently, Ukashi et al. reported that patients with quiescent small-bowel CD who had a retained PC had an increased risk of intestinal surgery, endoscopic dilation, and hospitalization [53]. Similarly, our study demonstrated that small-bowel patency containing PC evaluation was the most effective predictive factor for subsequent surgery in patients with newly diagnosed CD, with an auROC of 0.89. Among nominal covariates, ‘extensive small-bowel lesions’ was the second most effective predictive factor based on total inclusion counts. Therefore, this prediction model using covariates may be helpful in determining the likelihood of patients with newly diagnosed CD requiring surgery, which can lead to appropriate treatment for high-risk patients. For instance, the clinicians and patients can be prepared for the subsequent surgery and decide to opt for early ‘top-down’ therapeutic approaches to avoid or delay the surgery, although a surgery must be performed promptly in the case of a clinically absolute indication of surgery.

Nevertheless, this study had several limitations. First, this was a single-center, retrospective study with a minimum 1-year follow-up and a small dataset, although this study was restricted to newly diagnosed CD. Therefore, multicenter studies are warranted in the future. Second, we did not adopt the clinical scores at diagnosis, endoscopic scores, or laboratory activity markers as risk factors in this study since we aimed to make the model as simple as possible. Moreover, CD activity markers, such as C-reactive protein, erythrocyte sedimentation rate, blood albumin level, fecal calprotectin, leucine-rich a2-glycoprotein, Crohn’s Disease Activity Index (CDAI), and the simple endoscopic score for CD (SES-CD), as the endoscopic scores using CS are generally used; however, sometimes they may not accurately reflect the activity of CD (e.g., the development of infection, the risk of underestimating lesions in the proximal small intestine) [54]. Third, out of the four variables, small-bowel patency classification, major lesions, and extensive small-bowel lesions have substantial or a certain degree of overlap in clinical practice. This might affect the results. Fourth, the logistic regression model was fitted and validated using a small dataset without external validation. Additionally, the brute force method is often used when the problem space is sufficiently small or when there are no known algorithms or techniques to solve the problem more efficiently. Therefore, it is typically not the most efficient approach for complex problems but can serve as a baseline or fallback solution when other methods are not applicable or available.

## 5. Conclusions

The logistic regression model constructed using the brute force method to predict the risk of subsequent surgery in patients with newly diagnosed CD can enable clinicians and patients with CD to be prepared for the subsequent surgery and might be helpful in determining the first treatment. This system may increase the likelihood of an appropriate treatment strategy in patients with CD and contribute to improving their long-term quality of life.

## Figures and Tables

**Table 1:** Patient characteristics.

Characteristics	Overall
Sex (male:female)	69:24
Age (years)	26 (8–67)
Disease location	
Ileal type (L1)	18 (19)
Colonic type (L2)	21 (23)
Ileocolonic type (L3)	54 (58)
Disease behavior	
Non-stricturing, non-penetrating (B1)	50 (54)
Stricturing (B2)	35 (38)
Penetrating (B3)	8 (9)
Poor prognostic factor	
Young onset (<18 years old)	22 (24)
Major lesions (longitudinal ulcer and/or cobblestone appearance)	65 (70)
Upper small-bowel lesions	24 (26)
Extensive small-bowel lesions	32 (34)
Anal lesions	31 (33)
Body weight loss	73 (78)
Smoking	18 (19)
Small-bowel patency classification	
Group 0 (Passed PC)	65 (70)
Group 1 (Failed PC)	5 (5)
Group 2 (PC unavailable)	23 (25)
Required surgery	14 (15)

Data are presented as numbers (percentages) or medians (ranges). PC: patency capsule.

**Table 2:** A univariate comparison of covariates between patients with and without subsequent surgery.

		Surgery	Without Surgery	
	Covariates	n = 14	n = 79	*p*-Value
Continuous or discrete covariates (mean ± standard deviation)	Age (years)	35.36 ± 12.96	26.72 ± 11.99	0.038
	Poor prognosis score	3.43 ± 1.05	3.43 ± 1.83	0.996
	Small-bowel patency classification	1.50 ± 0.82	0.38 ± 0.75	<0.001
Nominal covariates (Checked counts/frequency)	Sex (Male)	10/0.71	59/0.75	0.751
	Young onset (<18 years of age)	1/0.07	21/0.27	0.175
	Ileal type	7/0.50	11/0.14	0.005
	Colonic type	2/0.14	19/0.24	0.729
	Ileocolonic type	5/0.36	49/0.62	0.082
	Main lesions	14/1.00	51/0.65	0.009
	Upper small-bowel lesions	1/0.07	23/0.29	0.105
	Extensive small-bowel lesions	9/0.64	23/0.29	0.015
	Anal lesions	2/0.14	29/0.37	0.130
	Body weight loss	12/0.86	61/0.77	0.726
	Smoking	4/0.29	14/0.18	0.461

Nominal covariates are expressed as checked counts or frequency; the differences between those with or without second injury were evaluated using Fisher’s exact test.

## Data Availability

The data presented in this study are available upon reasonable request from the corresponding author. The data are not publicly available due to privacy and ethical restrictions.

## Supplementary Materials

The following supporting information can be downloaded at: https://www.mdpi.com/article/10.3390/diagnostics13233587/s1, Table S1: Characteristics of the 14 patients who required surgery.
