# Supplementary material for: A Logistic Regression Model for Predicting the Risk of Subsequent Surgery among Patients with Newly Diagnosed Crohn’s Disease Using a Brute Force Method
Source: Diagnostics (Basel). 2023 Dec 3;13(23):3587. doi: 10.3390/diagnostics13233587 (PMC10706147; doi:10.3390/diagnostics13233587)
Supplement: Supplementary file 1 [file diagnostics-13-03587-s001.zip › diagnostics-2697047-supplementary.pdf]

TableS1. Characteristics of the 14 patients who required surgery.

Sex	Age	Disease location	HBI	Major lesions	Upper small-bowel lesions	Extensive small-bowel lesions	Anal lesions	Body weight loss	Smoking	Small-bowel patency classification	Initial treatment
M	36	Ileocolonic	5	+	+	-	-	+	-	2	IFX
M	18	Ileal	7	+	−	+	+	−	−	0	ED, 5-ASA, AZP, PSL,IFX
M	54	Ileal	13	+	−	+	−	+	−	2	Surgery
M	32	Ileal	7	+	−	+	−	+	+	2	ED, 5-ASA, IFX
F	29	Ileal	12	+	−	+	−	+	−	2	ED, 5-ASA, PSL, IFX
M	19	Ileocolonic	7	+	−	−	−	+	−	0	ED,5-ASA, IFX
F	18	Ileocolonic	15	+	−	+	+	+	−	2	Surgery
M	49	Ileal	2	+	−	+	−	−	+	2	Surgery
M	32	Ileal	3	+	−	+	−	+	−	2	ED, 5-ASA
M	42	Colonic	10	+	−	−	−	+	−	2	ED, PSL, IFX
M	20	Ileocolonic	5	+	−	+	−	+	+	2	ED, AZP, BUD, IFX
F	50	Ileal	2	+	−	−	−	+	−	2	ED, 5-ASA, BUD, UST
F	54	Colonic	17	+	−	−	−	+	+	1	ED, 5-ASA, PSL,VDZ
M	42	Ileal	1	+	−	+	−	+	−	0	ED, 5-ASA, ADA

HBI: Harvey-Bradshaw index, IFX: Infliximab, ED: Elemental diet, 5-ASA: 5-Aminosalicicylic acid,
AZP: Azathioprine, PSL: Prednisolone, UST: Ustekinumab, VDZ: Vedolizumab, ADA: Adalimumab
